# Etiology, Symptoms, and Treatment of Epilepsy: Advances and Perspectives

**DOI:** 10.3390/brainsci16010089

**Published:** 2026-01-14

**Authors:** Shampa Ghosh, Jitendra Kumar Sinha

**Affiliations:** 1 GloNeuro, Sector 107, Vishwakarma Road, Noida 201301, India; g.shampa17@gmail.com; 2 Symbiosis Institute of Health Sciences (SIHS), Symbiosis International (Deemed University), Pune 412115, India

Epilepsy is a chronic neurologic disorder characterized by recurrent and spontaneous seizures (Figure 1). It is known to affect more than 50 million people worldwide [1]. That is why it is considered one of the great healthcare challenges faced today [2]. It is a heterogeneous disorder, resulting from an extremely diverse range of genetic, structural, metabolic, and immune-mediated etiologies that have significant variations in terms of both clinical symptoms and therapeutic outcomes [3,4]. This Special Issue in the journal *Brain Sciences*, titled “Etiology, Symptoms, and Treatment of Epilepsy,” discusses several findings that have enhanced our knowledge of the disease process and provide potential opportunities for novel interventions [4,5,6]. Epilepsy is an etiologically complex disease [7,8]. Genetic generalized epilepsy exemplifies how complex polygenic influences and network dysfunctions contribute to the disease risk [9]. Additionally, it involves the thalamocortical circuits, which adds to therapeutic complications [7,9,10]. This is supported by emerging neuromodulation studies that target the anterior thalamic nuclei to modulate these circuits for subtypes of drug-resistant epilepsy [3,5].

Alongside genetic causes, structural lesions due to developmental malformations, trauma, infections, or stroke are still significant contributors to epilepsy [7,11]. On the other hand, neuroimaging advances offer better diagnosis and surgical candidacy evaluation [12,13]. Various neuroimmune mechanisms underlying metabolic dysfunction (like Type 2 diabetes mellitus) are known to promote seizure propensity [2,4]. Regarding this connection, Phoswa and Mokgalaboni have discussed the involvement of inflammatory cytokines like IL-1β, IL-6, and TNF-α, disruption of the blood–brain barrier, and oxidative stress in both the pathomechanisms of epilepsy and diabetes [14]. Neuroinflammation has gained increasing recognition as a central contributor to epileptogenesis and pharmaco-resistance [15,16,17]. It is also known to work through the mediation of activated microglia, astrocytes, and cytokine cascades [18,19,20]. This paradigm shift heralds immunomodulation as a promising adjunct therapeutic strategy [14,21,22].

Clinical Symptomatology

Epilepsy has a very wide symptomatology. It ranges from the seizures themselves to autonomic, cognitive, psychiatric, and behavioral dysfunction [4,6,23,24]. Salluce et al. provide compelling evidence of interictal autonomic dysfunction in pediatric epilepsy patients, identified by reduced skin conductance, which may underlie increased risks of SUDEP [6]. This finding implicates systemic autonomic involvement as part of epilepsy’s phenotypic spectrum, warranting holistic clinical assessment. Seizure semiology continues to be varied, including focal aware/impaired awareness seizures, as well as generalized tonic–clonic, myoclonic, and absence seizures, which all require different approaches, both diagnostically and therapeutically [2,4,25]. Ambulatory electrocorticography (including the closed-loop responsive neurostimulation) is revolutionizing the diagnostic yield and precision treatment [5,26,27]. Nevertheless, treatment adherence is crucial for controlling symptoms and improving quality of life [28,29]. Indeed, compliance, or lack thereof, remains a concerning issue that, intrinsically, is linked with socioeconomic class, lifestyle, and access to care, particularly among patients with reported alcohol use or unstable employment [30]. Treatment should be performed under regular clinical follow-up and patient education to optimize adherence and outcomes [31].

Therapeutic Innovations

Conventional ASMs can control seizures in the majority of patients, but a third go on to develop drug-resistant epilepsy [4,32]. Therefore, it is crucial to accelerate the process of development and integration of advanced therapies:

(a) Responsive Neurostimulation (RNS) for refractory generalized epilepsies targets the anterior thalamic nuclei, as described by the case series in this Special Issue [5,33]. The reduction in seizures and significant improvement in quality of life are worth highlighting [34]. This is a form of closed-loop neuromodulation that disrupts pathological network synchronization, heralding a personalized era of implantable therapies.
(b) Vagus Nerve Stimulation (VNS) and Deep Brain Stimulation (DBS) are neuromodulation modalities that modulate limbic and thalamic networks, thus offering supplementary seizure control in refractory cases [4,14,35,36]. Newer closed-loop variants of these devices hold promise for greater specificity and tolerability.
(c) Pharmacological and dietary adjuncts, including cannabidiol and ketogenic diets, have shown promise in specific refractory syndromes [37,38]. This area of research is especially encouraging in terms of non-pharmacologic complementary methods [2,4,39].
(d) Immunomodulatory therapies have helped to advance our understanding of neuroinflammatory mechanisms, charting innovative pathways for new treatments aimed at cytokines, inflammasomes, and glial activation [12,21,22]. These therapies have opened a new frontier of precision medicine where newly emerging fields can facilitate highly personalized, mechanism-based interventions. These would include genomic profiling, gene therapy, and optogenetics, especially for the monogenic forms of epilepsy.

Psychosocial Aspects of Epilepsy Care

In addition to the treatment mode, optimal management extends beyond pharmacological treatment and surgical interventions [40]. This is to address the multifaceted psychosocial determinants of disease trajectory, treatment adherence, and overall quality of life. According to Jopowicz et al. and Niriayo et al., these include lifestyle choices, social support systems, mental health comorbidities, societal stigma, and patient empowerment [31,41]. Lifestyle factors (including sleep hygiene, levels of stress, substance use, diet, and exercise) bear significantly on seizure control and adherence to treatment regimens [42,43]. Poor sleep hygiene, for example, is a recognized seizure precipitant; indeed, studies have shown that poor sleep hygiene is associated with increased seizure frequency [44,45]. Many patients do not appreciate the role that stress and fatigue may play in lowering seizure thresholds [46]. Encouraging lifestyle changes, engaging in stress management, and maintaining regular sleep is crucial to greatly reducing seizure burden.

Education about epilepsy increases self-efficacy and acceptance of the disease. This should be focused toward the eradication of myths and the decreasing of stigma [47]. Patients with knowledge regarding seizure triggers, the importance of medication, and safety management will have a higher chance of adhering to prescribed regimens [48]. Individualized psychoeducation sessions conducted by multidisciplinary teams of neurologists, psychologists, and social workers enable patients to understand and become more involved in the treatment program, providing them with more control over their disease, which decreases the anxiety associated with unpredictable seizures. Depression, anxiety, and social isolation are common comorbidities among people with epilepsy [49], and this is often understood to further worsen seizure control and impair psychosocial functioning [49,50]. Various psychological interventions like cognitive-behavioral therapy (commonly known as CBT) might improve mood and have a direct, positive effect on seizure frequency by reducing stress via adaptive coping styles [51]. The identification and management of psychiatric comorbidities improve not only mental health but also compliance and treatment outcome.

Nevertheless, the stigma associated with epilepsy remains a significant obstacle to social integration, employment, and education in many low-resource populations [52]. Negative perceptions often bring about the discrimination and social exclusion of people with epilepsy. This, too, contributes to ever-worsening depression and anxiety. Community-based awareness programs, as well as advocacy and policy reform, can serve to dispel misconceptions and improve the social acceptance of people affected by epilepsy [47,53]. Family members are very important in ensuring that medication is managed properly and that safety is monitored, as well as in providing emotional support [54]. Caregiver education programs that emphasize practical skills and psychosocial support have the potential to reduce caregiver burden and improve patient and family outcomes [55]. Promotion of open family discussions positively fosters trust and encourages adherence. Models like this are based on personalized treatment programs considering biological, psychological, and social needs. Importantly, these programs also need to take into consideration the peculiarities of an individual’s problems [56]. Increasingly, telehealth and digital tools have been able to provide seamless support, education, and self-management, especially in geographically remote or otherwise underserved areas [57].

Continued research into the psychosocial determinants of epilepsy underlines the need for evidence-based interventions. These need to be tailored to specific cultural, socioeconomic, and demographic contexts. We hope that future policy initiatives will be directed towards integrating mental health, creating community awareness, and assuring equal access to comprehensive care. A holistic, patient-centered approach promises a reduction in seizure frequency, an improvement in treatment program adherence, the empowerment of patients, the dismantling of societal stigma, and, ultimately, improved quality of life.

## Figures and Tables

**Figure 1:**
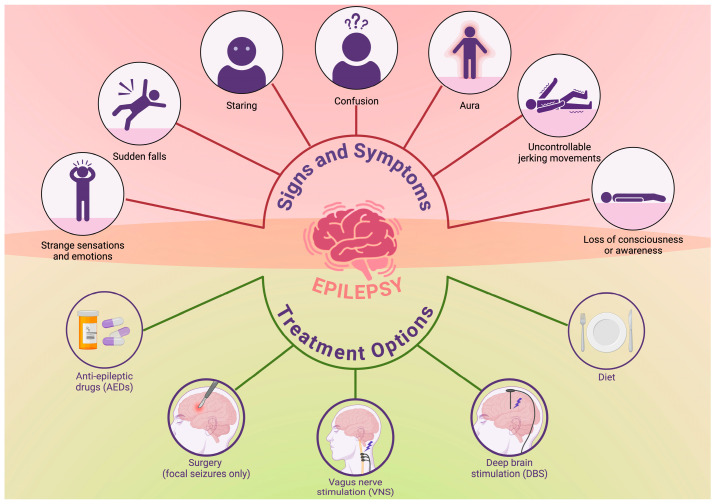
The signs and symptoms of epilepsy and the treatment options available. Created in BioRender. Singh, D. (2025). https://BioRender.com/3k45g59.

## Data Availability

No new data were created.
